# Hydrogen Sulfide Adsorption by Iron Oxides and Their Polymer Composites: A Case-Study Application to Biogas Purification

**DOI:** 10.3390/ma13214725

**Published:** 2020-10-22

**Authors:** Camilla Costa, Matteo Cornacchia, Marcello Pagliero, Bruno Fabiano, Marco Vocciante, Andrea Pietro Reverberi

**Affiliations:** 1 DCCI, Department of Chemistry and Industrial Chemistry, Università degli Studi di Genova, Via Dodecaneso 31, 16146 Genova, Italy; camilla.costa@unige.it (C.C.); matteo.cornacchia@unige.it (M.C.); marcello.pagliero@unige.it (M.P.); marco.vocciante@unige.it (M.V.); 2 DICCA, Department of Civil, Chemical and Environmental Engineering, Polytechnic School, Università degli Studi di Genova, Via Opera Pia 15, 16145 Genova, Italy; brown@unige.it

**Keywords:** hydrogen sulfide, biogas, adsorption, fixed bed, breakthrough curve, green technology

## Abstract

An experimental study of hydrogen sulfide adsorption on a fixed bed for biogas purification is proposed. The adsorbent investigated was powdered hematite, synthesized by a wet-chemical precipitation method and further activated with copper (II) oxide, used both as produced and after pelletization with polyvinyl alcohol as a binder. The pelletization procedure aims at optimizing the mechanical properties of the pellet without reducing the specific surface area. The active substrate has been characterized in its chemical composition and physical properties by X-ray Diffraction (XRD), Field Emission Scanning Electron Microscopy (FE-SEM), thermogravimetric analysis (TGA) and N_2_ physisorption/desorption for the determination of surface area. Both powders and pellets have been tested as sorbents for biogas purification in a fixed bed of a steady-state adsorption column and the relevant breakthrough curves were determined for different operating conditions. The performance was critically analyzed and compared with that typical of other commercial sorbents based on zinc oxide or relying upon specific compounds supported on a chemically inert matrix (SulfaTreat^®^). The technique proposed may represent a cost-effective and sustainable alternative to commercial sorbents in conventional desulphurization processes.

## 1. Introduction

Hydrogen sulfide (H_2_S) is one of the major concerns in civil activities [1] and industrial processes [2] where it represents an undesired compound for many different reasons. It poses serious safety challenges at a small and large scale owing to accidental releases, whose critical conditions are rapidly reached in case of fugitive emissions in confined or semi-confined spaces [3]. In open environments, landfills represent a considerable source of this compound [4], which is released into the atmosphere as a product of sulfate anion reduction carried out by bacteria. 

The presence of sulfur compounds in combustibles (fuels) is subject to progressively stringent constraints both for reasons related to environmental protection and also considering their secondary corrosive effects on metals, including stainless steel. The inherent safety approach, currently extended also to novel emerging processes [5], would require applying the guideword “substitution” to the fuel, however technical, economic and strategic constraints can strongly influence the actual achievement of this option [6]. Mercaptans, organic (poly) sulfides and thiophene derivatives are examples of sulfur-carrying molecules typically present in crude oil fractions, which undergo hydrodesulfurization processes producing H_2_S. In the first thermal step of the well-known Claus process, H_2_S is partially converted to SO_2_, while in the second catalytic step H_2_S reacts with the as-formed SO_2_ giving elemental sulfur [7].

Many technical solutions can be adopted for H_2_S abatement in gas purification technology depending on the specific compositions of the gas stream. Watanabe [8] critically analyzed the aforementioned technologies for natural gas desulphurization, usually relying upon liquid absorption, slurry reactors and fixed-bed columns. Alkylamine absorbents for sour gases [9] are typically adopted in large facilities [10] and they are economically demanding for high operating costs [11], together with drawbacks related to limitations in the selectivity and progressive degradation of the liquid sorbent. Slurry reactors [12] allow a good solid-fluid material exchange at relatively low operating temperatures and may represent, even in terms of sorbent recovery, a valid alternative to fixed-bed reactors. The latter came to prominence for their recent technological improvements, which make them a real cornerstone in H_2_S capture especially in the decontamination of biogas, a promising and renewable energy source deriving from anaerobic digestion of organic wastes. 

In their review, Andriani et al. [13] compared a biogas deriving from anaerobic digestion (AD) with the one produced from landfilling (LA). Notwithstanding a substantial similarity in terms of heating power, the differences in ammonia (NH_3_) and H_2_S concentration are remarkable, with H_2_S content for AD-biogas even 40 times greater than for LA-biogas. The maximum allowed concentration of residual H_2_S after biogas purification depends on its end use and in many cases, it falls down to a range of 0.5–10 ppm when biogas is intended for fuel cell feeding [14]. Higher concentrations may trigger a rapid electrode deterioration with a dramatic performance loss.

The solid sorbents adopted in fixed bed reactors for H_2_S capture may operate according to two different mechanisms, namely physisorption, by which weak bonds between H_2_S and the substrate are established and chemisorption, based on chemical bonds between the adsorbate and the substrate [15]. The latter is generally made of various composite materials based on metal foams [16], zeolites [17], laterites [18], kaolin [19], silica [20], carbon of miscellaneous sources [21] and other solid phases activated with proper metal salts and their mixtures in order to enhance the sorption capability of the fixed bed. Piergrossi et al. [22] investigated a case of impregnated active carbon (IAC) of commercial production, based on a ternary Cu-Cr-Fe combination of transition elements, for biogas purification. They argued that sulfur abatement by IAC can be ascribed to a complex sequence of steps, starting with a partial catalytic oxidation of H_2_S carried out by oxygen, whose role is fundamental in this kind of process having an optimal thermal set point at T = 130 °C. 

The need for milder operative conditions in order to fulfill more restrictive protocols related to process safety and energy savings, together with economic considerations [23], led to expanding the research towards sorbents like transition metal oxides operating at the lowest temperatures compatible with satisfactory chemisorption kinetics, but this task remains an open challenge. Weinlaender et al. [24] compared the performances of ZnO, a mixture of MnO-CuO and CuO-doped zeolite at different space velocities, H_2_S inlet concentrations and temperatures to test the influence of such variables on the global efficiency of H_2_S capture from synthetic biogas. Yang et al. [25] used the wet impregnation technique followed by calcination to prepare a sorbent made of ZnFe_2_O_4_ on activated carbon, having an H_2_S breakthrough capacity of 122.5 mg/g even at room temperature. Zahid et al. [26] investigated the synergistic effects and the sorption mechanism of different binary and ternary compounds made of metal oxides obtained by the coprecipitation method followed by calcination in some cases. Intriguingly, the best results in terms of adsorption capacity at ambient temperature were obtained for mixtures prepared without calcination. 

Nanochemistry and nanotechnology [27], with a new generation of smart materials [28], offer new opportunities in the use of elements [29] and compounds for a wide range of applications in physics and engineering [30], including gas purification. In this context, nanosorbents and nanocomposites [31] represent an attractive variant of the previously cited solid matrices as they benefit both from a high specific surface and enhanced overall kinetics of sulfur capture, but they often need careful control of the operative conditions during the synthesis of the sorbent phase. In the case of nanostructured iron oxides, such a disadvantage is more than offset by their absence of toxicity [32], together with a satisfactory efficiency at T < 100 °C and a good on-site regenerability. Many iron oxides of different compositions have been tested at room temperature and the role of local surface chemistry has been investigated in a recent study by Cao et al. [33], who pointed out the influence of oxygen vacancies and hydroxyl groups in H_2_S capture.

Head losses in a gas stream along a fixed bed is an important technical issue and it may become crucial for nanosized substrates. That is why many researchers focus their attention on the realization of sorbents having a high surface area together with a macroscopic granular structure possibly preserving these characteristics during both sorption and regeneration cycles. In this regard, some authors tested the use of composites made of polymers as binders for the solid active matrix. In some cases, the resulting pellet was not subject to calcination [34], while in others the binder underwent thermal dissociation at moderate temperatures in order to reach a good trade-off between mechanical properties and microporosity [35]. 

The goal of the present paper is to investigate the performances of two hematite-based sorbents for H_2_S removal, differing from each other about the presence or absence of a binder used for pelletization of the powder. The choice of iron (III) oxide aims at combining a satisfactory sulfur loading at low temperatures with ease of recycling, eco-compatibility and economy, the latter both in terms of production and operating costs. The paper is organized as follows. In Section 2, the process of sorbent synthesis is described and some details concerning its variants are given, together with the experimental set-up for biogas desulphurization. In Section 3, the obtained results are presented and compared with the performances attained by analogous sorbents discussed in the literature. In Section 4, the conclusions are drawn and possible future developments are outlined.

## 2. Materials and Methods

### 2.1. Reagents and Preparation of Sorbents

The following reagents: iron (III) chloride hexahydrate (FeCl_3_⋅6H_2_O, 98%, Sigma Aldrich, Milano, Italy), sodium hydroxide (NaOH, 98%, La Farmochimica, Genova, Italy), ammonia solution (NH_4_OH, 30%, Sigma Aldrich, Milano, Italy), copper (II) oxide (CuO, 99%, Fisher Scientific, Milano, Italy), polyvinyl alcohol (PVA, (C_2_H_4_O)*_n_*, 72 ± 10 kDa, 98%, La Farmochimica, Genova, Italy) were used as purchased and diluted, when necessary, with Milli-Q^®^ water. 

The methods adopted for the synthesis of hematite rely upon hydrolysis and precipitation of iron hydroxide (Fe(OH)_3_) using NaOH or NH_4_OH as bases in an aqueous medium. In both cases, Fe(OH)_3_ underwent further thermal treatments to reach the final product corresponding to α-hematite.

#### 2.1.1. Fe(OH)_3_ Synthesis by NaOH

A 30% NaOH solution was added dropwise to a 0.18 M FeCl_3_ solution in water kept under vigorous stirring at room temperature, obtaining a brick-red precipitate according to the Equation:

FeCl_3_ + 3 NaOH → Fe(OH)_3_ + 3 NaCl (1)

The solid phase was then separated by filtration and repeatedly washed with water to eliminate NaCl formed by Equation (1), which was carried out at three different pH values (10, 11 and 12) in order to investigate the effects of pH on the final product and its H_2_S removal capacity. After filtration, the precipitate was dried at 100 °C for 7 h, in order to desorb water from the solid phase. Such drying step was then followed by calcination, where Fe(OH)_3_ is firstly converted to goethite (α-FeOOH) and akaganeite (β-FeOOH) as indicated below:

Fe(OH)_3_ → FeOOH + H_2_O (2)

At higher temperatures, a dehydroxylation process led to the formation of α-hematite according to the Equation:

2 FeOOH → Fe_2_O_3_ + H_2_O (3)

Despite the repeated washing of Fe(OH)_3_ synthesized by Equation (1), a presence of residual NaCl was detected in the calcined powder. A further dispersion of the calcined phase in deionized water for 30 min at 80 °C, followed by filtration, drying at 100 °C and finally by calcination, led to obtaining a powder of α-hematite free from NaCl. The aforementioned solid phases resulting from progressive drying have been checked for the composition according to the characterization techniques described in the following Section 2.2 and Section 3.1.

#### 2.1.2. Fe(OH)_3_ Synthesis by NH_4_OH

This technique differs from the previous one in that it uses NH_4_OH in place of NaOH during the first step operated at 80 °C as follows:

FeCl_3_ + 3 NH_4_OH → Fe(OH)_3_ + 3 NH_4_Cl (4)

This variant of the Fe(OH)_3_ synthesis offers the advantage of avoiding the presence of NaCl in the final product obtained by calcination, as NH_4_Cl dissociates without leaving any solid residue. The remaining phases are the same as in the NaOH process.

#### 2.1.3. Pelletization of the Sorbent

The production of pellets is essential for a simpler execution of the industrial operations in real plants, where the recovery of the sorbent and its further regeneration are at a premium [34]. A first attempt consisted of realizing a pellet comprising a polybutadiene spherical core coated by a layer of sorbent located only at the surface of such an organic matrix. Such method, producing a pellet capable of adsorbing only superficially, was discarded in favor of a more reliable and proven technique relying upon mixing Fe_2_O_3_ with PVA as a binder, which was chosen for the following favorable features:

- the hydroxyl groups present in the molecular structure of PVA can interact with metal oxides/hydroxides by hydrogen bonds, thus enhancing the binding strength between active adsorbent and organic substrate whose reciprocal affinity is a basic parameter in polymer composites [36];
- PVA is insoluble in nonpolar solvents and it is soluble in water, thus allowing the preparation of aqueous solutions of controlled density and viscosity without resorting to toxic or expensive organic solvents;
- PVA itself is biocompatible and biodegradable under aerobic and anaerobic conditions. Such characteristics make it largely used in environmentally sound materials for a wide range of applications [37].

**Figure 1:**
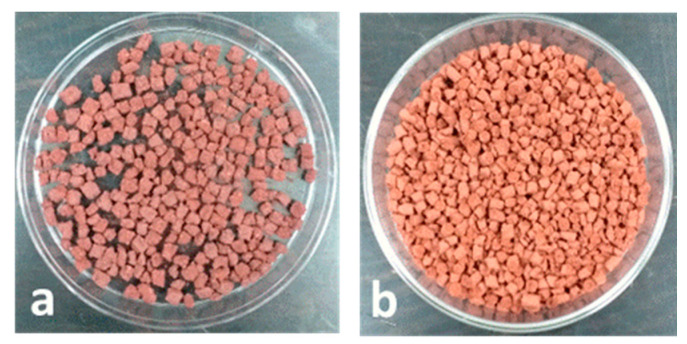
(**a**) Fe_2_O_3_-based pellets in square blocks. (**b**) Fe_2_O_3_-based pellets in cylinders.

The pelletization procedure required a preliminary qualitative screening of the rheological properties of ternary Fe_2_O_3_-PVA-H_2_O mixtures. The iron oxide-based adsorbent tends to flocculate in a paste containing less than 2.5% PVA, owing to a low viscosity of the medium. On the other hand, a concentration higher than 10% PVA gives gummy and unworkable pastes. For these reasons, a satisfactory trade-off was reached adopting a dispersion containing 5% PVA, which is suitable to be flattened in thin layers or extruded in strings after mixing with the hematite powder. Small blocks or cylinders (Figure 1) can be cut and dried reaching this final composition: 95% (*w*/*w*) Fe_2_O_3_ and 5% (*w*/*w*) PVA.

As in commercial products such as SulfaTreat^®^ [38], copper (II) oxide (CuO) was added to Fe_2_O_3_ in some samples during the preparation of pellets to enhance the adsorption capacity of the solid phase [39]. The copper oxide was selected among other possible metal oxides as its sorbent properties towards H_2_S are well known in the literature [40]. Three different kinds of pellets were produced containing 5%, 10% and 15% *w*/*w* CuO in the mixture, respectively. 

### 2.2. Characterization Techniques

Iron oxides modifications over temperature changes were determined by thermal gravimetric analysis using a thermogravimetric analysis (TGA)/DSC1 STAR instrument Mettler-Toledo, Greifensee, Switzerland). The analyses were conducted in an air stream (80 mL/min), at a heating rate of 10 °C/min.

The morphology and surface composition of the sorbent was determined by a Field Emission-Scanning Electron Microscope (FE-SEM, Zeiss Supra 40 VP, Karl Zeiss SMT, Oberkochen, Germany). The sample was deposited on a polycarbonate Millipore Isopore filter with 200 nm pore size for data acquisition carried out in three different modes, namely Energy Dispersive X-ray Spectrometry (EDS), secondary electrons (SE) collected by an in-lens detector and backscattered electrons (BSE). Observations were conducted at different magnifications up to 500,000× and the accelerating voltage was fixed at 20 kV.

The crystalline phases of the sorbent and its three-dimensional structure were characterized by X-ray Diffraction (XRD) using a Philips X Pert MPD diffractometer with a radiation source (CuKα, λ = 1.54 Å) operating at 40 kV, a scanning angle 2θ in a range 5–95° at a time step of 3 s. The surface area and pore size distribution of iron oxide powder and pellets were determined by Accelerated Surface Area Porosimetry (ASAP 2020 Plus, Micromeritics Ltd., Hexton, UK), using N_2_ as adsorbed probe gas. The surface area *A*_s_ is calculated according to the expression:

(5) $$A_{s}=(V_{m}/V_{mol})N_{a}\sigma /m$$

where *V_m_* is the monolayer capacity of adsorption, *V_mol_* is the standard N_2_ molar volume, *N_a_* is the Avogadro number, *σ* is the molecular cross-sectional area occupied by an N_2_ adsorbate molecule and *m* is the mass of the sample.

All the reported results were obtained as an average of at least three measurements, with a relative standard deviation not exceeding 5%.

### 2.3. The Experimental Set-up for Desulphurization Tests

**Figure 2:**
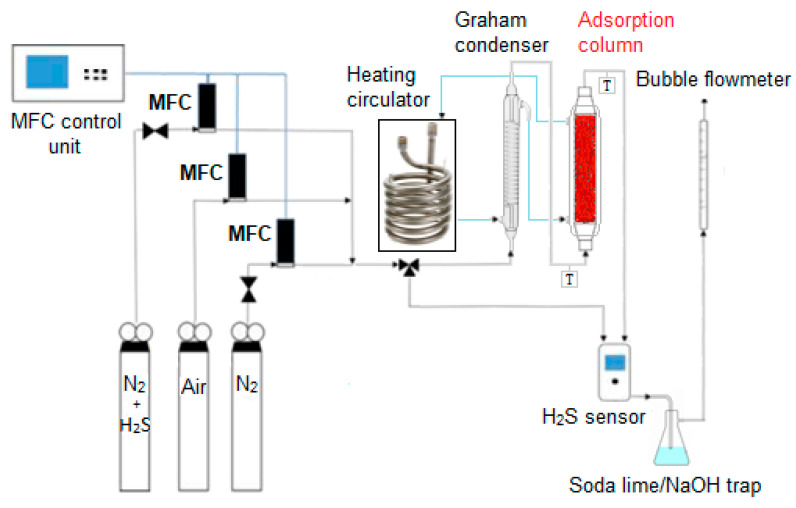
Schematic representation of the adsorption plant.

The sorption tests for powder and pellets were carried out using the apparatus schematized in Figure 2. Three pressurized gas cylinders containing respectively 1000 ppm H_2_S in N_2_, air and N_2_ are connected with a three-way joint to obtain a gas mixture at the selected H_2_S concentration for reactor feed, whose flow rate and temperature are tuned by a mass flow controller (MFC) for each cylinder and by a Graham condenser equipped with an external water jacket powered by a heating circulator (F12-MA, Julabo, Milano, Italy). The latter controls also the temperature of the adsorption column, whose dimensions are:

- 8 cm length and 0.8 cm diameter for tests on powder;
- 10 cm length and 2.5 cm diameter for tests on pellets.

All tests were carried out at atmospheric pressure. The sorption temperatures were intentionally kept at T ≤ 50 °C for iron oxides powders and pellets in order to minimize running costs, which were mainly related to an increasing power consumption for growing temperatures. On the other side, capital costs depend primarily on the physico–chemical properties of the sorbent, on the cost of equipment and plant construction materials needing high corrosion resistance towards H_2_S at high temperatures. An inherently friendlier process design requires fewer control instruments and protective equipment, thus implying lower lifetime costs including capital costs, regular control system testing and maintaining costs.

The concentration of H_2_S, both at the column inlet and outlet, was monitored by a digital sensor (Crowcon Detection Instruments Ltd., Oxfordshire, UK) operating in a range of 0–200 ppm with a ±2 ppm standard deviation. An automatic bubble flowmeter measures the input and output flow from the adsorption unit. A soda-lime or NaOH trap for tail gases was adopted to avoid the dispersion of unadsorbed H_2_S in the atmosphere.

## 3. Results and Discussion

### 3.1. Iron Oxides Powders

**Figure 3:**
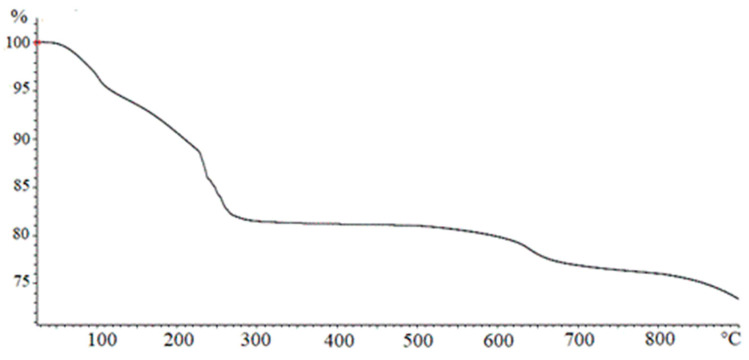
Thermogravimetric analysis (TGA) analysis on a Fe(OH)_3_ sample.

In Figure 3, a TGA analysis of Fe(OH)_3_ obtained by NaOH according to the method described in Section 2.1.1 is reported. The curve shows four different regions corresponding to the different loss of weight percentage occurring at growing temperatures. In the first phase, when T < 100 °C, the adsorbed water was removed. The second weight loss, for 100 < T < 230 °C, can be ascribed to the elimination of hydration water described by Equation (2), leading to the formation of goethite. 

The third weight loss, for 230 < T < 270 °C, may be referred to as the transformation of goethite into hematite, as indicated by Equation (3). A slight weight loss detected at T > 600 °C can be attributed to a particle sintering with a structural transition from micropores to macropores resulting in a final water loss.

**Figure 4:**
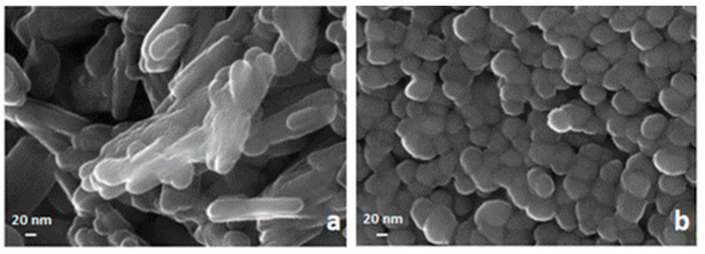
FE-SEM images of a nanosized solid phase obtained by calcination at 400 °C of Fe(OH)_3_ synthesized by precipitation with NaOH (**a**) and by precipitation with NH_4_OH (**b**).

An FE-SEM analysis of two samples of hematite prepared respectively by precipitation of Fe^3+^ with NaOH at pH = 12 and with NH_4_OH at pH = 8 are reported in Figure 4. Both of them were obtained by calcination at 400 °C. The sample by NaOH visualized in the left panel appears to be formed by bundles of nanorods somewhat randomly oriented, while the one obtained by NH_4_OH shows a dense agglomerate of spheroidal nanometric particles with a low degree of polydispersity.

A definitive confirmation about the structure and chemical composition of the solid phases obtained by thermal dissociation of Fe(OH)_3_ comes from XRD analysis. A first XRD spectrum was registered for a powder of FeOOH obtained from the NaOH method and dried at 100 °C for 8 h. Despite a strong background noise due to the presence of a considerable amount of amorphous solid, the observed peaks satisfactorily match with the ones of α-FeOOH (goethite) taken from the Inorganic Crystal Structure Database (ICSD). 

**Figure 5:**
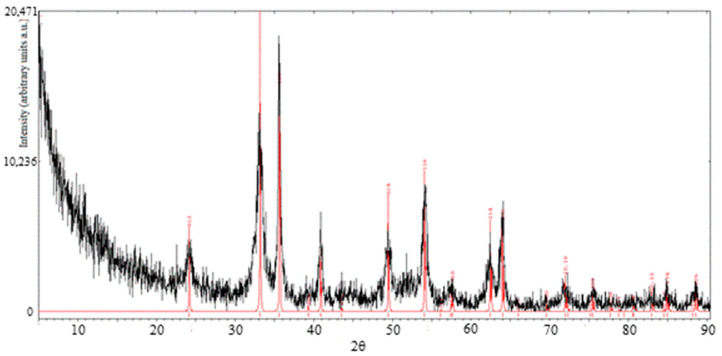
XRD patterns of hematite sample (black curve) and reference hematite (red curve).

In a second XRD analysis, a sample of Fe_2_O_3_ was investigated. It was produced from the NaOH method, calcined at 300 °C for 30 min and washed with deionized water at 80 °C to remove NaCl salt. The results are shown in Figure 5. The sample spectrum is reported in black color. The reference peaks, in red, are those of the hematite, ICSD 64599. 

The pattern of the sample is consistent with the one of reference hematite (α-Fe_2_O_3_) and the peaks typical of NaCl are not observable, thus proving the effectiveness of the washing procedure carried out after calcination. 

### 3.2. Adsorption Tests on Hematite Powders

Some preliminary tests on hematite powder, here not reported for the sake of brevity, were carried out at room temperature and pressure in order to determine the optimal parameter settings aiming at maximizing the sulfur capacity of the sorbent. It was ascertained that hematite synthesized by Equation (4) gives an exceedingly fine powder leading to unacceptable head losses when packed in the column. For this reason, hematite produced by NH_4_OH was discarded from further H_2_S adsorption tests and only hematite synthesized by NaOH at pH = 12, calcined at 300 °C for 30 min and finally washed at 80 °C in deionized water will be considered in the following experimental tests, both for powders and pellets. 

**Figure 6:**
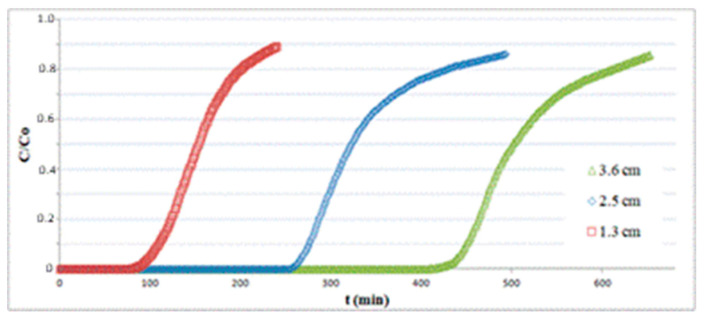
Adsorption tests on hematite powder with different bed lengths.

Figure 6 depicts the breakthrough curves at T = 25 °C for three different bed lengths obtained with a superficial velocity of 0.14 m/s and an inlet H_2_S concentration of 150 ppm. The amount of H_2_S adsorbed per unit mass of substrate *q* at a fixed time *t* is calculated as follows:

(6) $$q=\frac{Qy_{in}PW_{H_{2}S}}{m_{b}RT}\int_{0}^{t}\left(1-C/C_{0}\right)dt$$

where *Q* is the total volumetric flow rate, *P* is the pressure, *y_in_* is the H_2_S molar fraction at the column inlet, *W*_H2S_ is the molar mass of H_2_S, *m_b_* is the mass of the bed, *R* is the gas constant, *T* is the temperature of the gaseous stream, *C* is the outlet H_2_S concentration and *C*_0_ is the inlet H_2_S concentration. 

As expected, the breakpoint time *t_b_* (selected at *C*/*C*_0_ = 0.1) grows for increasing values of bed length, but the adsorption capacity varies only slightly, as reported in Table 1. 

**Figure 7:**
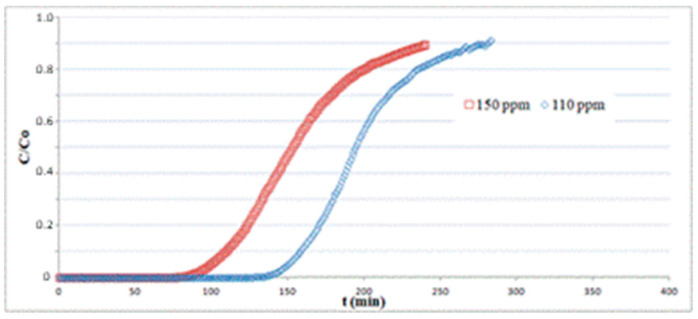
Adsorption tests on hematite powder with different inlet H_2_S concentrations at T = 25 °C.

In Figure 7, tests for different inlet H_2_S concentrations *C*_0_ at 110 and 150 ppm were carried out on a 1.3 cm-length bed at a gas velocity of 0.14 m/s and T = 25 °C. The data for *C*_0_ = 150 ppm are the same as indicated in Table 1. The breakpoint time gets higher for decreasing values of inlet H_2_S concentrations, as the fixed bed requires longer times to reach the saturation for smaller values of H_2_S partial pressure in the column feed. The sorption capacity *q*, obtained using Equation (6) by numerical integration of the corresponding plots, does not show significant variations, ranging from *q* = 21.2 at *C*_0_ = 150 ppm to *q* = 20.7 at *C*_0_ = 110 ppm. 

### 3.3. Adsorption Tests on Hematite Pellets

The adsorption tests on pellets were carried out to ascertain whether the hematite embedded in a polymeric matrix, mixed or unmixed with CuO as a promoter, keeps the performances typical of powders themselves. The performance of pellets manufactured with the technique previously described in Section 2.1.3 was compared with that typical of two well-known commercial sorbents for H_2_S, namely zinc oxide pellets (ZnO, supplied by Iplom S.p.A., Busalla, Italy) and SulfaTreat^®^ 410 HP pellets. In particular, the latter was investigated by Di Felice and Pagliai [41], who tested the performances of such sorbent under the same operative condition here adopted. All pellets realized in the present study use hematite powders prepared according to the procedure described at the beginning of Section 3.2.

A preliminary TGA analysis (whose plot is not reported here) proved that PVA undergoes a slow thermal degradation at T ≅ 200 °C, while its dissociation kinetics speeds up considerably at T = 280 °C. For these reasons, T < 200 °C in the adsorption column can be considered as a precautionary criterion when PVA is adopted as a pellet binder. 

The surface area of the as-prepared pellets was investigated to ascertain the effect of the binder on this variable. In Table 2, the results of Brunauer-Emmett-Teller (BET) based calculations for pellets of various compositions are reported, together with the data pertaining to powders for comparison. Such results show that the addition of PVA to powders may significantly alter the surface area, but the order of magnitude is preserved. On the other hand, the PVA concentration in the pellet was kept at the lowest possible value to ensure the rheological properties needed for the paste to be extruded. 

**Figure 8:**
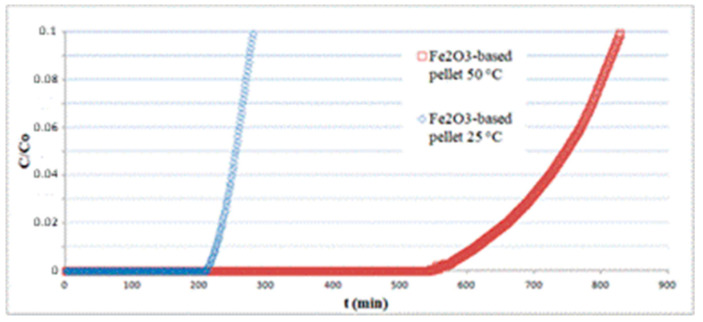
Effect of temperature on the performance of a hematite-based pellet bed.

The pellets, having a cylindrical shape with approximately 2.5 mm diameter and 3.2 mm height, were randomly packed in a fixed bed of 7 cm length and tested for H_2_S adsorption at different temperatures. All experiments were carried out using a total inlet gas flow rate of 810 mL/min containing 150 ppm H_2_S. In Figure 8, the results concerning Fe_2_O_3_-based pellets at 25 and 50 °C are reported. The sorption curves are limited to relatively short times, as an informative experiment on the whole breakthrough curve would require it to be continued overnight, with serious safety issues. The results show that temperature has a considerable effect on the sorption capacity, varying from *q* = 4.1 at 25 °C to *q* = 12.1 at 50 °C, both calculated by integration of the curves at *t* = *t_b_*(*C*/*C*_0_ = 0.1).

A more complete picture of the global scenario in terms of sorption capacity at different temperatures for pellets of various composition can be obtained by inspection of Table 3, whose data refer to a breakpoint time corresponding to *C*/*C*_0_ = 0.01 to compare the present results with literature data, including the ones taken from [41] concerning SulfaTreat^®^. At room temperature, even the poorer performance offered by Fe_2_O_3_ pellets prepared according to the method proposed in this study is greater than the ones obtainable by ZnO or SulfaTreat^®^ pellets in the same operating conditions. Concerning ZnO, a possible explanation could be given reminding that, in many previous investigations, ZnO proved to be kinetically active only at temperatures much higher than the ones here considered [42]. Another reason could stem from differences in surface area, estimated at 54 m^2^/g for Fe_2_O_3_ versus 23 m^2^/g for ZnO pellets. The lower sorption capacity of SulfaTreat^®^ pellets concerning hematite pellets could be ascribed to a difference in intrinsic sorption mechanisms, occurring only at the surface of SulfaTreat^®^ pellets, acting exclusively through a thin external layer.

Inferring an intrinsic synergy between CuO and Fe_2_O_3_ embedded in pellets is a more difficult task. At a first analysis, the beneficial effects of CuO on Fe_2_O_3_ could be simply motivated by the different sorption capacity of the pure substances. In this case, the hypothesis that each oxide works irrespective of the other seems to be the more reasonable, as only a rough mixing of powders was performed during the pellet preparation. 

### 3.4. Preliminary Regeneration Tests 

Regeneration and recycling/disposal of the exhaust adsorbent are fundamental issues to be faced to realize a “zero waste” process, as required by a green and sustainable approach. According to the open literature, the regeneration of iron sulfides has been widely studied at high temperatures (500−900 °C) in the field of gas refinery [43]. In the biogas sector, regeneration at low temperatures is still a partially unexplored topic and complete and/or reliable information is scarce.

Some preliminary regeneration tests were performed on hematite pellets with air at ambient temperature. Possible products are Fe_2_O_3_, SO_2_ and elemental S.

At the end of the adsorption phase on a virgin bed, a swapping air stream, with a 410 mL/min flow rate, was passed on the bed for 5 h at 25 °C. The goals were to explore the regeneration degree of the adsorbent after this soft treatment and to gain some insight into the regeneration products. The first question was to ascertain whether SO_2_, produced by the action of air on the fixed bed, could be present downstream. To this purpose, an H_2_S sensor and a soda-lime trap were connected in series to the reactor output. The H_2_S sensor was added to detect any hydrogen sulfide leakage, while the soda-lime trap was designed to capture any gaseous acid possibly formed. During the selected time of 5 h, the H_2_S sensor did not record any presence of hydrogen sulfide, while the soda-lime trap showed a color shift revealing the presence of SO_2_ in the gas stream. As a consequence, there is no risk of releasing harmful H_2_S once the bed is put in contact with an air stream. 

Another preliminary assessment was carried out on a loaded bed extracted from the reactor and left in contact with ambient air for 30 days. At the end of this period, another H_2_S removal test was performed to evaluate the effectiveness of the regeneration. 

**Figure 9:**
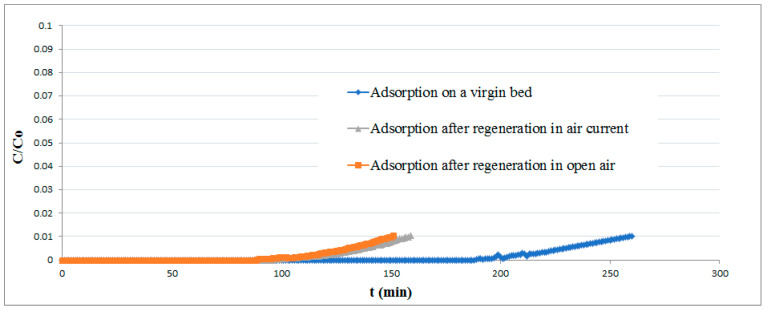
Breakthrough curves for the virgin and regenerated adsorbent.

The results of experimental runs performed on both fresh and regenerated pellets are reported in Figure 9. The difference in the adsorption curves makes it clear that the regenerated bed lost a part of its removal efficiency, probably owing to elemental sulfur deposition on the pellet surface. 

About 68% of theoretical efficiency can nevertheless be achieved, a much higher value than that obtained by previous investigations [44]. No improvement in the regeneration performances was achieved by increasing the exposure time of the bed to air.

As a further technical option, the effect of pure oxygen or oxygen-enriched air at different regeneration temperatures could be investigated, but this option would lead to an increase in the total costs of the process, introducing an additional hazard as well.

These preliminary results seem to suggest that regeneration is efficient three or four times before a new bed is needed. On the other hand, at the end of their lifetime, the pellets proposed in this study can be disposed of without being considered hazardous waste. 

## 4. Conclusions

In this paper, novel techniques for the synthesis of hematite-based sorbents for H_2_S capture from biogas are proposed and experimentally tested. From a methodological point of view, the most important achievements can be summarized in the following points:

- Hematite represents a positive choice as a base for sorbents to be used in H_2_S abatement from biogas, as it minimizes both capital and production costs.
- The outlined process highlights the opportunity of choosing a route under mild conditions, thus combining substitution and attenuation roads to inherently safer plants.
- To the best of our knowledge, PVA as a binder represents a novelty in the field of iron oxides-based adsorbents. It proved to be useful for all powder blends here adopted, owing to its adhesive properties, ease of utilization and biodegradability.
- The pellets produced and tested at room temperature in the present study showed a sorption capacity higher than the one typical of commercial products, under the same operating conditions.
- The pelletization process here proposed, based on a minimum necessary amount of binder in the pellet, represents a satisfactory trade-off between the improvement of mechanical properties and the preservation of surface area needed for a good adsorption efficiency.
- Preliminary tests on sorbent regeneration have shown that iron (III) oxide pellets may regain satisfactory adsorption efficiency even adopting regeneration cycles by air at room temperature.

Future investigations will be addressed to the use of binders made from products of biological origin to further improve the sustainability of the process here described.

## Figures and Tables

**Table 1:** Adsorption performance of hematite powder as a function of bed length at T = 25 °C.

Bed Length (cm)	Residence Time (s)	Break Point Time (s)	q Adsorbed at C/Co = 0.1 (mg/g)	q Adsorbed at C/Co = 0.9 (mg/g)
1.3	0.38	109	21.2	28.2
2.5	0.73	276	23.4	29.1
3.6	1.1	421	24.7	30.4

**Table 2:** BET results for the surface area of pellets and powders.

Sample	Surface Area (m^2^/g)
Fe_2_O_3_ powder	105
CuO powder	35
Fe_2_O_3_ pellet	54
Fe_2_O_3_ + 5% CuO pellet	52
Fe_2_O_3_ + 10% CuO pellet	49
Fe_2_O_3_ + 15% CuO pellet	47

**Table 3:** Summary of pellet properties and their comparison with other commercial sorbents. The values concerning SulfaTreat^®^ have been calculated using the data taken from [41].

Sample (Pellets)	Break Point Time (min)	q Adsorbed at C/C_0_ = 0.01 (mg/g)
Fe_2_O_3_ (25 °C)	225	3.9
Fe_2_O_3_ (50 °C)	610	9.1
Fe_2_O_3_ + 5% CuO (25 °C)	265	4.1
Fe_2_O_3_ + 10% CuO (25 °C)	315	4.7
Fe_2_O_3_ + 15% CuO (25 °C)	435	6.6
ZnO (25 °C)	237	2.4
ZnO (50 °C)	304	3.2
ZnO (80 °C)	344	3.6
SulfaTreat^®^ 410 HP (25 °C)	150	2.5
